# PET/CT Imaging Characteristics of Gastric-Type Endocervical Adenocarcinoma: Findings from a Small Exploratory Series

**DOI:** 10.3390/curroncol32100530

**Published:** 2025-09-23

**Authors:** Yun Wang, Xuan Zhou, Yun Xi, Hanmei Lou, Linfa Li, Heqing Yi, Tao Zhu

**Affiliations:** 1 Department of Nuclear Medicine, Zhejiang Cancer Hospital, Hangzhou 310022, China; wangyun@zjcc.org.cn (Y.W.); pet-ct001@163.com (L.L.); 2 Postgraduate Training Base Alliance, Zhejiang Cancer Hospital, Wenzhou Medical University, Hangzhou 310022, China; zhouxuan2025@sina.com; 3 Department of Pathology, Zhejiang Cancer Hospital, Hangzhou 310022, China; xiyun@zjcc.org.cn; 4 Department of Gynecological Radiotherapy, Zhejiang Cancer Hospital, Hangzhou 310022, China; louhm@zjcc.org.cn; 5 Department of Gynecological Oncology, Zhejiang Cancer Hospital, Hangzhou 310022, China

**Keywords:** cervical adenocarcinoma, diagnosis cervical gastric-type adenocarcinoma, 18F-FDG PET/CT, squamous cell carcinoma, usual-type endocervical adenocarcinoma, diagnosis

## Abstract

Objective: To identify distinctive 18F-FDG positron emission tomography (PET)/computer tomography (CT) features of gastric-type endocervical adenocarcinoma (GAS) that differentiate it from squamous cell carcinoma (SCC) and usual-type endocervical adenocarcinoma (UEA), as well as to correlate these findings with pathological characteristics. Methods: Patients treated between December 2018 and December 2024 were retrospectively reviewed. The study included 12 GAS, 48 SCC, and 30 UEA cases. Evaluated parameters included tumor morphology, cystic components, uterine cavity fluid, N/M staging, tumor diameter, the cervical lesion maximum standardized uptake value (SUVmax), and the tumor-to-liver maximum standardized uptake ratio (T/L SUVmax). Results: GAS predominantly exhibited diffuse infiltrative growth (11/12), in contrast to mass-like growth observed in SCC (37/48) and UEA (24/30) (both *p* < 0.001). Cystic components, uterine cavity fluid, and peritoneal metastasis occurred significantly more frequently in GAS (12/12, 11/12, 5/12, respectively) compared to SCC and UEA (all *p* < 0.001). Elevated CA19-9 levels were more common in GAS (9/12) compared with SCC (*p* < 0.001). Tumor diameter did not differ significantly among the groups (*p* > 0.05). SUVmax and T/L SUVmax values were significantly lower in GAS (7.5 ± 3.8 and 2.5 ± 1.6, respectively) than in UEA (19.1 ± 11.4 and 5.7 ± 3.4) and SCC (17.4 ± 6.7 and 5.5 ± 2.6) (all *p* < 0.001). Conclusion: The clinical characteristics of GAS include infiltrative tumor growth, fluid accumulation in the uterine cavity, frequent formation of microcystic or macrocystic components, peritoneal metastasis, and elevated CA19-9 levels. In this cohort, SUVmax and T/L SUVmax values in GAS were significantly lower than those observed in SCC and UEA.

## Simple Summary

Gastric-type endocervical adenocarcinoma (GAS), which is not associated with high-risk HPV infection, presents with atypical clinical features that result in low detection rates in screening and biopsy procedures. Its biological characteristics, particularly its aggressive behavior, present substantial challenges for both diagnosis and treatment. The preoperative diagnostic rate remains low, often leading to missed or incorrect diagnoses, thereby delaying treatment and adversely affecting patient prognosis. Although specific magnetic resonance imaging features of GAS have been identified, the relationship between positron emission tomography (PET)/computer tomography (CT) imaging characteristics and the pathological features of GAS is not well understood. This study demonstrated that PET/CT and clinical features of GAS include infiltrative tumor growth, fluid accumulation in the uterine cavity, frequent presence of microcystic or macrocystic structures, peritoneal metastasis, and elevated CA19-9 levels. The maximum standardized uptake value and tumor-to-liver maximum standardized uptake ratio for GAS were significantly lower than those for squamous cell carcinoma and usual-type endocervical adenocarcinoma. These findings may support early detection by emphasizing the diagnostic value of PET/CT features and the imaging–pathology correlation in GAS.

## 1. Introduction

Cervical cancer is the most common malignant tumor of the female reproductive system. The majority of cervical cancer cases are associated with persistent infection by high-risk human papillomavirus (HPV); however, certain subtypes, particularly specific cervical adenocarcinomas, are not linked to HPV. The International Endocervical Adenocarcinoma Criteria and Classification categorizes cervical adenocarcinomas into HPV-associated and non-HPV-associated subtypes [1,2]. The implementation of primary prevention strategies, including HPV vaccination and health education, along with secondary measures such as cervical cancer screening, is anticipated to significantly reduce the incidence of HPV-associated cervical cancer. Nevertheless, the proportion of cases unrelated to HPV is projected to increase gradually [3]. These non-HPV-associated tumors exhibit distinct epidemiological, clinical, pathological, and molecular genetic features, and are characterized by high malignancy, poor prognosis, and a high likelihood of being missed or misdiagnosed. Due to their substantial clinical impact, increased attention to these tumors is warranted.

Gastric-type endocervical adenocarcinoma (GAS) is the predominant subtype of non-HPV-associated cervical cancers and represents the second most common primary cervical adenocarcinoma following usual-type endocervical adenocarcinoma (UEA) [4]. GAS is a mucinous adenocarcinoma with gastric differentiation and exhibits morphological features resembling those of the pyloric gland epithelium. It arises independently of high-risk HPV infection and typically presents with atypical clinical manifestations. Lesions are often concealed, complicating tissue sampling and resulting in low positivity rates in both screening and biopsy procedures. Moreover, the pathological features of GAS can mimic benign conditions, while its biological behavior is highly aggressive, posing significant diagnostic and therapeutic challenges. The preoperative diagnostic rate is low, and GAS is frequently missed or misdiagnosed, leading to delayed treatment and adversely affecting patient outcomes [5]. Therefore, identifying the imaging characteristics of GAS and their correlation with pathological features is of critical importance.

Although certain imaging features of GAS—such as upper cervical segment involvement and the presence of cystic components—have been reported in the literature [1,6], the correlation between imaging characteristics and pathological findings remains insufficiently understood. This retrospective study analyzed positron emission tomography (PET)/computer tomography (CT) findings in patients with GAS and compared them with those of squamous cell carcinoma (SCC) and UEA. Distinctive PET/CT features that differentiate GAS from SCC and UEA were identified, and correlations between imaging and pathological features were elucidated.

## 2. Materials and Methods

### 2.1. Patient Selection

Consecutive patients with GAS who underwent PET/CT imaging at our institution between December 2018 and December 2024 were retrospectively enrolled. Inclusion criteria required a pathological diagnosis of cervical cancer, absence of prior treatment before imaging, and no history of other malignancies. Exclusion criteria included concurrent primary malignancies, pathological types other than GAS, SCC, or UEA, and incomplete clinical data. Pathological diagnoses were based on hematoxylin and eosin (H&E) staining to assess tumor morphology, following the 2020 World Health Organization Classification of Tumors of the Female Genital Tract [7]. Following histomorphological evaluation of H&E-stained sections, immunohistochemical staining was performed as needed, using the following antibodies: p16 (E6H4, prediluted; Roche, Basel, Switzerland), MUC6 (OTIR4C11, 1:100; Zhongshan Golden Bridge Biotechnology, Zhongshan, China), Claudin18.2 (LBP1-CLDM18, prediluted; Amoy Diagnostics, Xiamen, China), PAX8 (LBP2-PAX8, 1:200; Amoy Diagnostics, Xiamen, China), p53 (MX008, 1:200; MXB Biotechnologies, Fuzhou, China), estrogen receptor (ER) (SP1, prediluted; Roche, Basel, Switzerland), and progesterone receptor (PgR) (636, prediluted; Dako, Glostrup, Denmark). SCC and UEA cases with available PET/CT data from the same period were included for comparison with the GAS cases. Clinical and pathological characteristics—including age, pretreatment International Federation of Gynecology and Obstetrics (FIGO) stage (2018 criteria), 9th Edition of the American Joint Committee on Cancer stage, and serum tumor markers (CA19-9, CA125, SCC)—were obtained from medical records. The final study cohort comprised 12 patients with GAS, 48 with SCC, and 30 with UEA. This study was approved by the Ethics Committee of Zhejiang Cancer Hospital (IRB-2025-857).

### 2.2. 18F-FDG PET/CT Image Acquisition

This study adhered strictly to established guidelines for 18F-FDG PET/CT examinations [8]. The standardized protocol required patients to fast for 6–8 h before imaging, with blood glucose levels maintained at or below 8.3 mmol/L. An intravenous dose of 18F-FDG (3.7 MBq/kg) was administered, followed by a 60 ± 10-min rest period in a dimly lit environment. Before PET/CT scanning using GE Discovery PET/CT 710 and Siemens PET/CT Biograph Vision 600 systems, patients were instructed to empty their bladders and consume 500 mL of water to ensure adequate distension of the gastric lumen. Imaging extended from the vertex to the upper thigh. Dedicated acquisition protocols were employed, with each trunk bed position requiring 2–3 min. Concurrent CT scans were performed with parameters of 120 kV, 100 mA, and a 3 mm slice thickness. CT images were used for attenuation correction, and whole-body PET, CT, and PET/CT fusion images were reconstructed using appropriate algorithms (GE Discovery PET/CT 710: ordered-subsets expectation maximization [OSEM]; Siemens PET/CT Biograph Vision 600: Ultra HD). All post-processing was conducted using the corresponding workstations (GE Discovery PET/CT 710: Advanced Workstation AW4.6, GE Medical Systems, Waukesha, WI, USA; Siemens PET/CT Biograph Vision 600: Syngo.via).

### 2.3. Data Collection and Image Analysis

Two board-certified nuclear medicine physicians independently reviewed each PET/CT scan and reached a consensus on the imaging findings. The analysis included assessment of tumor growth patterns (mass-forming or diffuse infiltrative), presence and size of cystic components (none, microcyst ≤ 3 mm, or macrocyst > 3 mm), tumor diameter, maximum standardized uptake value (SUVmax), and the tumor-to-liver SUVmax ratio (T/L SUVmax). SUVmax of cervical lesions was determined by outlining the region of interest. Liver SUVmax was measured in a region with homogeneous FDG uptake within the right hepatic lobe, avoiding large vessels and abnormal parenchyma. The T/L SUVmax was calculated by dividing the SUVmax of the cervical lesion by the liver SUVmax. Tumors were classified as “mass-forming” if they demonstrated well-defined margins with outward expansion, and as “diffuse infiltrative” if they exhibited inward spread involving at least half of the cervix [9]. The presence of intrauterine fluid accumulation was also evaluated. All lesion evaluations were based on PET/CT fused images with a focus on CT morphological changes. For metabolically inactive infiltrative areas, localization was performed using the accompanying CT.

### 2.4. Statistical Analysis

**Figure 1:**
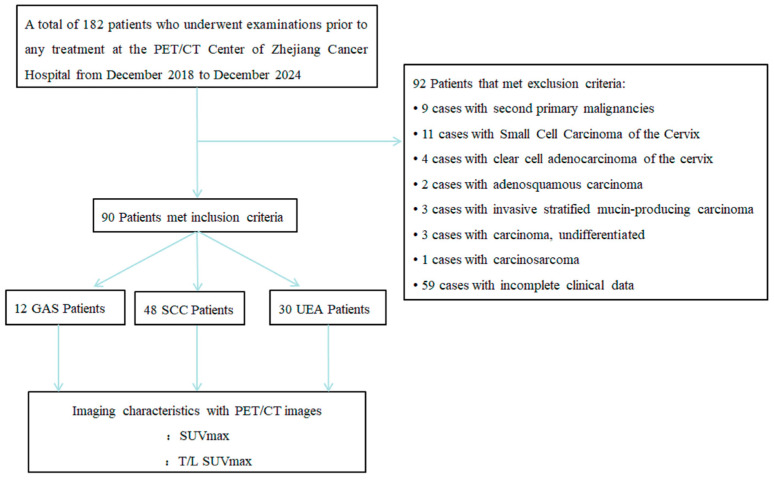
A schematic representation of the study workflow.

A schematic overview of the study workflow is presented in Figure 1. Statistical analyses were performed using R version 3.5.3 and SPSS version 27.0. Continuous variables were evaluated for normality using the Shapiro–Wilk test. Data with normal distribution were compared using Student’s t-test, whereas non-normally distributed data were analyzed with the Mann–Whitney U test. Categorical variables were assessed using either the chi-square test or Fisher’s exact test, as appropriate. All statistical tests were two-tailed, and a *p*-value of less than 0.05 was considered statistically significant.

## 3. Results

### 3.1. Patient Characteristics

The study included 12 patients with GAS (median age: 63 years), 48 with SCC (median age: 56 years), and 30 with UEA (median age: 55 years). Table 1 summarizes the baseline clinical characteristics of the patients, including their FIGO stages.

### 3.2. PET/CT Imaging Characteristics

Table 2 provides a summary of PET/CT findings related to tumor morphology. Regarding tumor growth patterns, 1 case in the GAS group exhibited a mass-forming pattern, while the remaining 11 cases demonstrated diffuse infiltrative growth. This distribution differed significantly from that in the SCC and UEA groups (*p* < 0.001). Intrauterine fluid accumulation was observed in 11 of 12 GAS cases, a frequency significantly higher than that seen in the SCC group (11 cases; *p* < 0.001) and the UEA group (12 cases; *p* = 0.0024). The presence of cystic components varied markedly among the three groups. In the GAS group, 1 case exhibited microcysts and 11 cases showed macrocysts. In contrast, only 9 cases in each of the SCC and UEA groups showed either microcysts or macrocysts. Peritoneal metastases were identified in 5 of 12 GAS cases, significantly more frequent than in the SCC group (0 cases; *p* = 0.0001) and the UEA group (0 cases; *p* = 0.0009). The distribution of peritoneal metastases among the 5 GAS patients was as follows: right subdiaphragmatic area (1 case), omentum (5 cases), right paracolic gutter (2 cases), left paracolic gutter (2 cases), bowel mesentery/serosa (4 cases), and pelvic peritoneum (1 case).

There were no statistically significant differences in N-stage among the GAS, SCC, and UEA groups (*p* > 0.05). Likewise, no significant difference in M-stage was observed between the GAS and SCC groups (*p* = 0.0528); however, a significant difference was found between the GAS and UEA groups (*p* = 0.0061).

**Figure 2:**
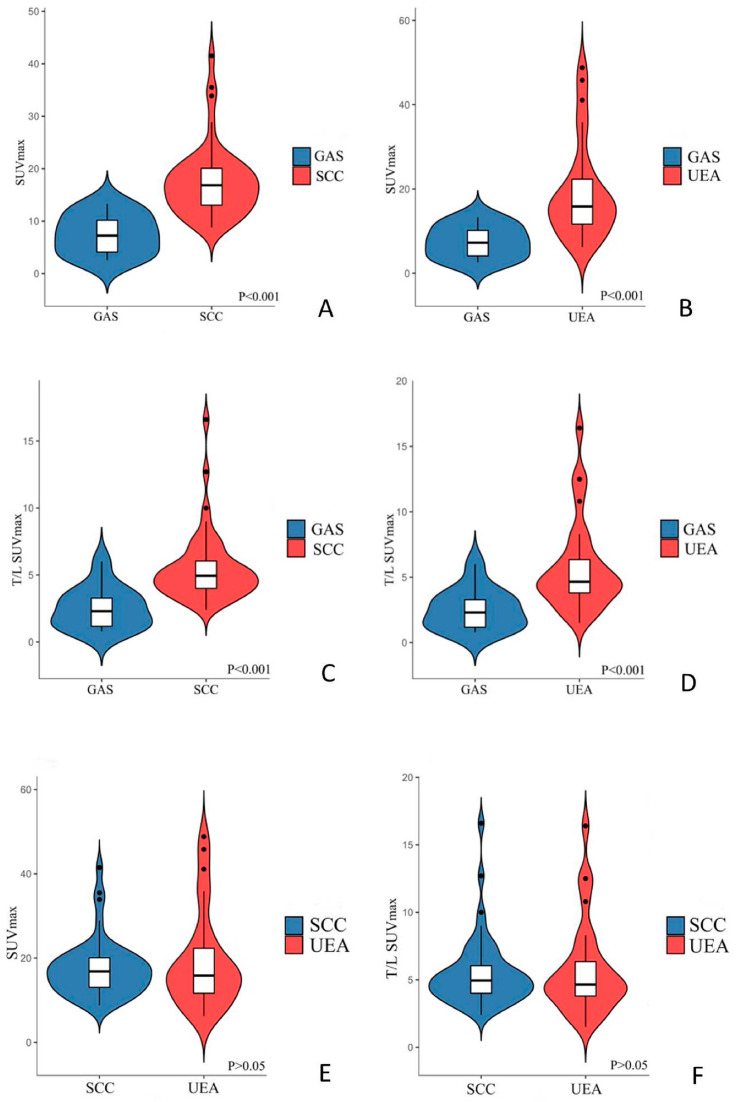
Violin plots illustrating the distribution of SUVmax values in GAS versus SCC (**A**), GAS versus UEA (**B**), T/L SUVmax values in GAS versus SCC (**C**), GAS versus UEA (**D**), as well as SUVmax (**E**) and T/L SUVmax (**F**) comparisons between SCC and UEA.

Tumor diameter did not differ significantly among the GAS (4.4 ± 1.5 cm), SCC (4.8 ± 1.8 cm), and UEA (3.9 ± 1.5 cm) groups (*p* > 0.05). The SUVmax and T/L SUVmax values were significantly lower in the GAS group (7.5 ± 3.8 and 2.5 ± 1.6, respectively) compared with the UEA group (19.1 ± 11.4 and 5.7 ± 3.4) and the SCC group (17.4 ± 6.7 and 5.5 ± 2.6) (*p* < 0.05 for all comparisons). No significant differences were observed in SUVmax or T/L SUVmax between the SCC and UEA groups (*p* > 0.05 for both). These comparisons are illustrated in Figure 2.

**Figure 3:**
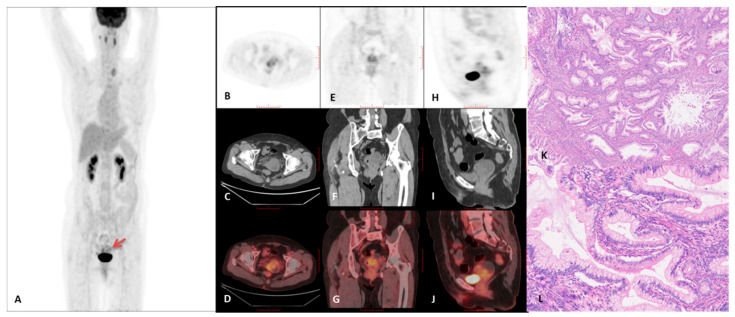
A case of a 62-year-old female with gastric-type adenocarcinoma, demonstrated by 18F-FDG PET/CT imaging. (**A**) Maximum intensity projection (MIP) PET image. (**B**–**D**) Axial PET, CT, and PET/CT fusion images. (**E**–**G**) Coronal PET, CT, and PET/CT fusion images. (**H**–**J**) Sagittal PET, CT, and PET/CT fusion images. The imaging showed intense 18F-FDG uptake within the cervical lesion (red arrow), with a maximum standardized uptake value (SUVmax) of 6.8 and a tumor diameter of 4.6 cm. The liver SUVmax was 3.9. Additionally, intrauterine fluid accumulation and a macrocyst were observed. (**K**) Histopathological analysis revealed irregular mucinous glands scattered within the cervical stroma (×40, H&E staining), with some glands exhibiting intraluminal papillary structures. (**L**) Further histological examination showed tumor cells with abundant clear or eosinophilic cytoplasm, distinct cell membranes, rare mitotic figures and apoptotic bodies, and moderate nuclear atypia (×200, H&E staining).

**Figure 4:**
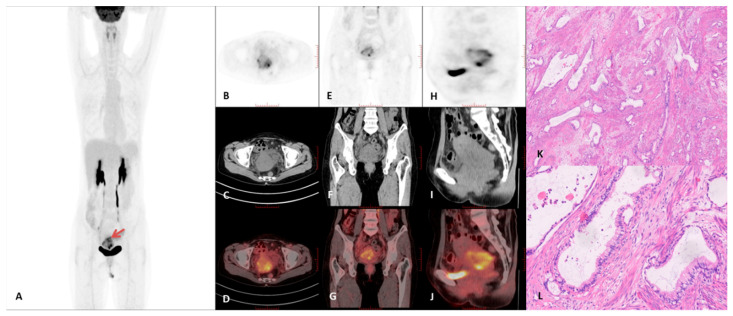
18F-FDG PET/CT imaging findings in a 55-year-old female diagnosed with gastric-type adenocarcinoma. (**A**) Maximum intensity projection (MIP) PET image**.** (**B**–**D**) Axial PET, CT, and PET/CT fusion images. (**E**–**G**) Coronal PET, CT, and PET/CT fusion images. (**H**–**J**) Sagittal PET, CT, and PET/CT fusion images. The imaging demonstrated markedly increased 18F-FDG uptake in the cervical lesion (red arrow), with a maximum standardized uptake value (SUVmax) of 13.3 and a tumor diameter of 5.9 cm. The liver SUVmax was recorded at 2.2. Intrauterine fluid collection was not observed in this patient; however, a macrocyst was present. (**K**) Histopathological examination revealed irregular mucinous glands haphazardly distributed within the cervical stroma (×40, H&E staining), with some glands showing angulation and distortion. (**L**) Higher magnification histology demonstrated tumor cells with abundant clear or eosinophilic cytoplasm, distinct cell membranes, rare mitotic figures, and apoptotic bodies. The nuclei exhibited moderate to severe atypia (×200, H&E staining).

**Figure 5:**
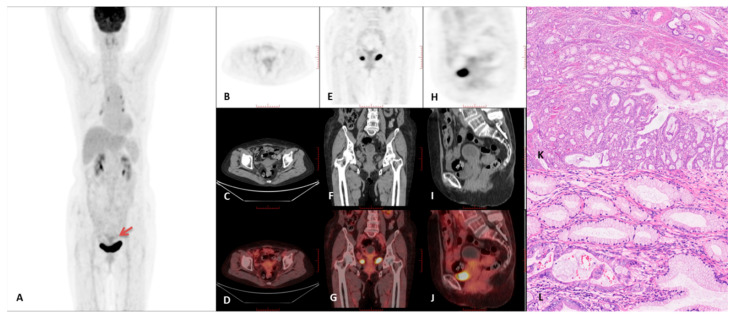
18F-FDG PET/CT imaging findings in a 77-year-old female diagnosed with gastric-type adenocarcinoma. (**A**) Maximum intensity projection (MIP) PET image. (**B**–**D**) Axial PET, CT, and PET/CT fusion images. (**E**–**G**) Coronal PET, CT, and PET/CT fusion images. (**H**–**J**) Sagittal PET, CT, and PET/CT fusion images. The PET/CT imaging showed mildly increased 18F-FDG uptake in the cervical lesion (red arrow), with a SUVmax of 4.5 and a tumor diameter of 3.7 cm. The liver SUVmax was 3.7. Intrauterine fluid collection and a macrocyst were also observed. (**K**) Histopathological examination revealed irregular mucinous glands dispersed throughout the cervical stroma (×40, H&E staining). (**L**) Histopathological analysis showed tumor cells with abundant clear or eosinophilic cytoplasm, distinct cell membranes, rare mitotic figures and apoptotic bodies. Nuclear features ranged from mild atypia (upper region) to moderate-to-severe atypia (lower left corner) (×200, H&E staining).

**Figure 6:**
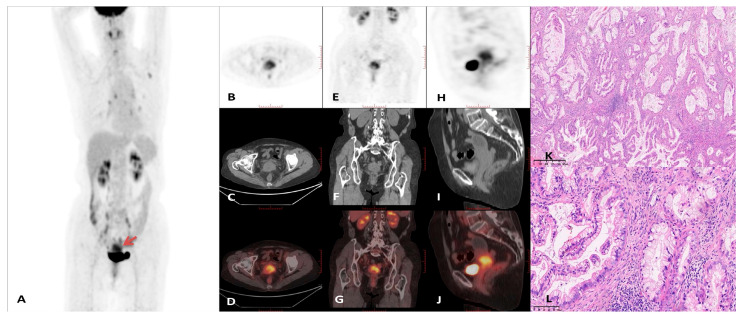
A 70-year-old female diagnosed with gastric-type adenocarcinoma, as demonstrated by 18F-FDG PET/CT imaging. (**A**) Maximum intensity projection (MIP) PET image. (**B**–**D**) Axial PET, CT, and PET/CT fusion images. (**E**–**G**) Coronal PET, CT, and PET/CT fusion images. (**H**–**J**) Sagittal PET, CT, and PET/CT fusion images. The imaging revealed intense 18F-FDG uptake in the cervical lesion (red arrow), with a SUVmax of 9.7 and a tumor diameter of 3.3 cm. The liver SUVmax was 3.6. Intrauterine fluid collection and a macrocyst were also present. (**K**) Histopathological examination showed irregular mucinous glands haphazardly arranged within the cervical stroma (×40, H&E staining), with some glands displaying intraluminal papillary formations. (**L**) High-power histology revealed tumor cells with abundant clear or eosinophilic cytoplasm, distinct cell membranes, and infrequent mitotic figures and apoptotic bodies. Nuclei showed moderate-to-severe atypia (×200, H&E staining).

Representative PET/CT images and corresponding histopathological findings are presented in Figure 3, Figure 4, Figure 5 and Figure 6. A comprehensive summary of PET/CT findings in the GAS group is provided in Table 3.

### 3.3. Immunohistochemical Characteristics of the GAS Group

Among the 12 GAS cases, p16 expression was positive in 6 cases and negative in 6. MUC6 was positive in all cases. Claudin18.2 was positive in 9 cases, with staining intensities ranging from + to +++, and negative in 3 cases. PAX8 expression was positive in 3 cases and negative in 9. p53 was positive in 8 cases and negative in 4. Estrogen receptor (ER) was positive in 1 case and negative in 11. Progesterone receptor (PR) was negative in all cases. Table 4 provides a detailed summary of the immunohistochemical findings for the GAS group.

## 4. Discussion

This retrospective study analyzed the PET/CT imaging features of patients with GAS and compared them to those of patients with SCC and UEA, with the aim of identifying distinguishing PET/CT characteristics of GAS. Based on the findings, GAS presents with at least five distinctive features: an infiltrative tumor growth pattern, frequent intrauterine fluid accumulation, the presence of microcysts or macrocysts, peritoneal metastasis, and elevated CA19-9 levels. Additionally, the GAS group demonstrated significantly lower SUVmax and T/L SUVmax values than the SCC and UEA groups, suggesting differences in tumorigenic mechanisms and histological features unique to GAS.

MRI scans of patients with GAS frequently reveal growth localized to the upper cervical region [10,11]. Since GAS is unrelated to HPV infection, it lacks the oncogenic mechanisms typical of HPV-associated tumors [12,13]. Known precursor lesions of GAS include lobular endocervical glandular hyperplasia (LEGH) and Peutz–Jeghers syndrome, with LEGH often localized in the upper endocervical canal [14,15,16]. These associations are consistent with the distinct tumorigenic pathways of GAS compared to HPV-associated carcinomas.

The diffuse infiltrative growth pattern appears to be a hallmark feature of GAS. In 2007, Kojima et al. [17] introduced the pathological term “gastric-type endocervical adenocarcinoma” and proposed that minimal deviation adenocarcinoma represents a well-differentiated form within the differentiation spectrum of GAS. Macroscopically, GAS is characterized by cervical hypertrophy with a smooth surface, and tumors typically arise in the mid to upper endocervical canal. They frequently measure approximately 4 cm in diameter and may present as a “barrel-shaped” cervix or as exophytic masses, such as cauliflower-like or firm nodular lesions. GAS also demonstrates pronounced local tumor infiltration, including full circumferential involvement of the endocervical canal, deep myometrial invasion extending to the serosal surface, and infiltration into the uterine body and parametrial tissues. Studies have indicated a higher frequency of TP53 mutations and epithelial–mesenchymal transition-associated genetic alterations in GAS compared to HPV-related tumors [4]. The invasive components of GAS often exhibit sparse glandular distribution relative to SCCs and most UEAs. Consequently, cystic lesions in GAS tend to be more prominent in superficial rather than deep stromal regions.

Diffuse infiltrative tumors such as GAS may remain undetected on PET/CT due to their relatively low metabolic activity, a pattern more commonly observed in GAS than in SCC. A notable PET/CT characteristic of GAS in this study was the high frequency of intrauterine fluid accumulation. Among the 12 GAS cases, 11 exhibited intrauterine fluid, which may be attributable to the tumor’s histological behavior. GAS commonly demonstrates endophytic, diffusely infiltrative growth, accompanied by stromal fibrosis that reduces the cervical tissue’s elasticity and narrows the endocervical canal. Although intrauterine fluid accumulation is not a direct feature of GAS, it is likely a secondary consequence of cervical rigidity and excessive mucin secretion [9]. As a relatively easily detectable feature on PET/CT, MRI, and CT, intrauterine fluid may serve as a valuable auxiliary indicator for identifying GAS.

The GAS group also showed a significantly higher incidence of macrocyst formation compared to SCC and UEA. This may reflect the tumor’s pronounced mucin-secreting capability [18,19]. In early disease stages, GAS displaces normal cervical architecture, including columnar epithelium of the endocervical canal and Nabothian cysts, contributing to the development of dilated cystic structures. Additionally, the secretion of copious mucus by tumor cells may result in mucus-filled cystic lesions within the cervix.

Histopathologically, GAS demonstrates features consistent with mucinous adenocarcinoma, with glandular epithelium resembling that of gastric foveolar, pyloric, or pancreatobiliary differentiation. Tumor cells contain abundant gastric-type mucin and exhibit both morphological and immunohistochemical evidence of gastric differentiation. Markers such as HIK1083 and MUC6 are useful for confirming gastric-type features. Other markers, including p16, p53, PAX8, ER, and PR, may provide additional diagnostic support for GAS [20].

This study also evaluated Claudin18.2 immunohistochemical expression in 12 GAS cases. Of these, 9 cases showed positive expression (ranging from + to +++), including 3 cases (Cases 3, 5, and 6) with strong positivity (+++), while 3 cases (Cases 1, 9, and 12) were negative. As a marker of gastric-type differentiation, Claudin18.2 expression may be influenced by tumor heterogeneity, differentiation status, or molecular subtype. Strong positivity (+++) is generally associated with more typical gastric-type differentiation and aligns with the presence of abundant mucin secretion and phenotypic gastric features in GAS. Conversely, negative expression may suggest subclonal variations or the presence of non-gastric-type differentiation components, reflecting the biological heterogeneity of GAS. Notably, the frequent and intense expression of Claudin18.2 in GAS suggests that these patients may be potential candidates for anti-Claudin18.2 targeted therapy. Furthermore, the expression level of Claudin18.2 may be associated with tumor invasiveness, metastatic potential, or therapeutic response. However, larger-scale studies and mechanistic investigations are necessary to further elucidate the clinical significance of Claudin18.2 in GAS.

GAS may be associated with elevated tumor markers. More than 50% of patients with GAS present with increased CA19-9 levels, while approximately one-third demonstrate elevated serum CA125 levels. Elevated CA125 is often indicative of pelvic or abdominal metastases, and a small proportion of patients may also exhibit elevated carcinoembryonic antigen (CEA) levels [21]. In this study, elevated CA19-9 and CA125 were observed in 9 of 12 and 6 of 12 GAS cases, respectively.

The analysis revealed no significant differences in tumor diameter and N-stage between the GAS group and either the SCC or UEA groups. Similarly, M-stage did not significantly differ between the GAS and SCC groups; however, a statistically significant difference was observed between the GAS and UEA groups. Furthermore, the GAS group demonstrated significantly lower SUVmax and T/L SUVmax values compared to both SCC and UEA groups. This finding can be attributed to the presence of mucinous cysts and the sparse tumor cell distribution within fibrotic stroma in solid-dominant tumors. For instance, as shown in Figure 5, tumors with limited glandular components embedded in extensive fibrotic stroma exhibited reduced SUVmax and T/L SUVmax values, which may hinder accurate diagnosis. Despite low metabolic parameter values, malignancy should still be considered when characteristic PET/CT features are observed.

To date, there have been no published studies—domestic or international—specifically describing the 18F-FDG PET/CT imaging manifestations of GAS. However, as demonstrated in this study, unlike the commonly encountered cervical SCC, GAS typically shows only partial FDG uptake within solid components, with most tumor regions displaying little to no significant FDG accumulation. This pattern may impact the ability to detect systemic metastatic lesions, particularly small-volume metastases. By integrating the non-HPV-associated status of GAS with its infiltrative growth, frequent intrauterine fluid accumulation, presence of microcysts or macrocysts, and propensity for peritoneal metastasis—together with PET/CT and pathological findings—clinicians may improve the early identification of GAS. Given its diagnostic difficulty and poor prognosis, the development of effective early detection strategies for GAS remains a critical need [22,23,24].

Additionally, this study found no significant difference in metabolic parameters (SUVmax and T/L SUVmax) between SCC and UEA (*p* > 0.05), indicating that 18F-FDG PET/CT is more effective in distinguishing GAS from other cervical cancer subtypes than in differentiating between SCC and UEA. This may be due to the high metabolic activity shared by both SCC and UEA, leading to similar glucose uptake profiles. Comparable tumor cell densities, proliferative capacities, and tumor microenvironments may also contribute to the observed overlap in PET/CT metabolic features. Consequently, reliance on metabolic indices alone may be insufficient for differentiating SCC from UEA. Comprehensive evaluation integrating morphological imaging features, immunohistochemical profiles (e.g., p16, p53), and clinical presentation remains essential for accurate classification.

In this study, a relatively high proportion of patients were diagnosed with stage IVB disease in the GAS (8/12), SCC (21/48), and UEA (8/30) groups. Several factors may contribute to this distribution. The study population was drawn from a Class A tertiary cancer hospital, which also functions as a National Cancer Regional Medical Center. Such institutions often treat advanced or complex cases, including patients with distant metastases. Delayed initial diagnoses or prolonged referral processes may have contributed to the advanced disease stage observed at presentation. In addition, the aggressive biological behavior of specific tumor subtypes—such as GAS—may inherently lead to more frequent advanced-stage diagnoses. Furthermore, as a retrospective study, there is potential for selection bias, with a greater likelihood of including patients with complete clinical records and long-term follow-up, many of whom may have had late-stage disease. Therefore, the distribution of stage IVB cases in this cohort may not reflect the true incidence in the general population, but rather the referral patterns specific to the study center.

It is also noteworthy that, in Table 3, Case 10 within the GAS group exhibited markedly elevated SUVmax and T/L SUVmax values compared to other GAS cases. This patient was classified as stage IVB, and postoperative pathology revealed metastasis in 1 of 5 right deep inguinal lymph nodes, along with tumor involvement in the fibroadipose tissue of the rectouterine pouch. Advanced-stage tumors often demonstrate increased metabolic activity, likely due to enhanced cell proliferation, hypoxia, and elevated glucose consumption. These features may explain the high SUVmax and T/L SUVmax values observed in this case.

This study offers several strengths relevant to cervical cancer research. First, it provides a systematic comparison of clinicopathological and metabolic characteristics across three major cervical cancer subtypes: GAS, SCC, and UEA. By incorporating PET/CT imaging findings with immunohistochemical markers such as Claudin18.2, the study highlights the value of integrating metabolic and molecular features to improve diagnostic accuracy and prognostic assessment. Second, the investigation offers detailed analysis of tumor growth patterns and their correlation with advanced clinical stage, contributing new insights into the invasive nature and progression mechanisms of GAS. All cases were drawn from a National Cancer Regional Medical Center, ensuring data integrity, standardized diagnostic protocols, and clinical applicability. The inclusion of both common and rare cervical cancer subtypes enhances the study’s novelty and scope. These advantages underscore the significance of this work in advancing understanding of cervical cancer heterogeneity and supporting the development of personalized diagnostic and treatment strategies.

This study has several limitations. First, due to the rarity of GAS, only 12 patients with GAS were included, resulting in a limited sample size that may affect statistical power and constrain detailed subgroup or secondary endpoint analyses. Second, PET/CT image acquisition occurred over an extended timeframe and involved multiple scanner models. Although standardized imaging protocols were followed, variations in image quality, acquisition settings, and reconstruction algorithms across devices may have influenced the consistency of both quantitative and qualitative assessments. Third, the limited spatial resolution of PET imaging presents challenges in reliably detecting small lesions, early stromal invasion, or micrometastases—particularly in cases of the diffuse infiltrative growth patterns characteristic of GAS—which may result in an underestimation of total tumor burden. Furthermore, as reported in recent multicenter studies, preoperative imaging demonstrates limited sensitivity in detecting parametrial invasion, vaginal involvement, and particularly lymph node and peritoneal metastases in GAS. As a single-center retrospective study, this analysis is also subject to selection bias. Therefore, validation through larger, multicenter studies is required to enhance generalizability.

## 5. Conclusions

Our findings indicate that GAS is characterized by an infiltrative tumor growth pattern, intrauterine fluid accumulation, frequent cystic formation, peritoneal metastasis, and elevated CA19-9 levels. In our cohort, the SUVmax and T/L SUVmax values in GAS were significantly lower than those observed in SCC and UEA. These distinct PET/CT features were consistent with pathological findings and support the diagnostic utility of PET/CT for GAS. Collectively, these insights may aid in improving the radiological diagnosis of cervical cancer and serve as a foundation for the development of early detection strategies for GAS.

## Figures and Tables

**Table 1:** Patient demographics in this cervical cancer study.

Characteristic		GAS Patients	SCC Patients	UEA Patients
Number		12	48	30
Age (years), median (range)		63 (29–77)	56 (25–74)	55 (28–75)
FIGO (2018), *n*	IB	1	13	0
	IIA	1	7	7
	IIIB	0	0	3
	IIIC	1	7	12
	IVA	1	0	0
	IVB	8	21	8

**Table 2:** Comparison of PET/CT findings between GAS, SCC, and UEA.

	GAS (N = 12)	SCC (N = 48)	UEA (N = 30)	*p*-Value	
				GAS vs. SCC	GAS vs. UEA
Growth pattern				<0.001	<0.001
Mass forming	1	37	24		
Diffuse infiltration	11	11	6		
Intrauterine fluid				<0.001	0.0024
Present	11	11	12		
Absent	1	37	18		
Cyst				<0.001	<0.001
Microcyst	1	0	0		
Macrocyst	11	9	9		
None	0	39	21		
N-stage				0.7334	0.5041
N0	5	20	12		
N1	1	9	10		
N2	6	19	8		
M-stage				0.0528	0.0061
M0	3	27	22		
M1	9	21	8		
Peritoneal metastasis				0.0001	0.0009
Present	5	0	0		
Absent	7	48	30		
tumor diameter mean value (cm)	4.4 ± 1.5	4.8 ± 1.8	3.9 ± 1.5	0.4678	0.3643
SUVmax	7.5 ± 3.8	17.4 ± 6.7	19.1 ± 11.4	<0.001	<0.001
T/L SUVmax	2.5 ± 1.6	5.5 ± 2.6	5.7 ± 3.4	<0.001	<0.001
Ca199 (U/mL)				<0.001	0.7186
≤37	3	41	11		
>37	9	7	19		
Ca125 (U/mL)				0.5081	0.292
≤35	6	31	9		
>35	6	17	21		
SCC (U/mL)				<0.001	0.09
≤1.5	10	6	16		
>1.5	2	42	14		

**Table 3:** PET/CT findings of each cervical gastric-type adenocarcinoma case.

Case #	FIGO 2018	Tumor Diameter Mean Value (cm)	Macrocysts	Tumor Growth Pattern	Intrauterine Fluid	SUVmax	T/L SUVmax
1	IVB	2.9	Present	Diffuse	Present	3.0	1.0
2	IVA	2.4	Present	Diffuse	Present	2.8	1.1
3	IB	3.7	Present	Diffuse	Present	2.6	0.8
4	IVB	6.5	Present	Diffuse	Present	9.3	3.9
5	IVB	3.3	Present	Mass	Present	9.7	2.7
6	IIIC	4.8	Present	Diffuse	Present	11.6	3.1
7	IIA	4.6	Present	Diffuse	Present	6.8	1.7
8	IVB	2.9	Present	Diffuse	Present	12.6	3.8
9	IVB	5.2	Microcysts	Diffuse	Present	6.2	1.9
10	IVB	5.9	Present	Diffuse	Absent	13.3	6.0
11	IVB	6.8	Present	Diffuse	Present	7.7	3.1
12	IVB	3.7	Present	Diffuse	Present	4.5	1.2

#: number.

**Table 4:** Immunohistochemical characteristics of each cervical gastric-type adenocarcinoma case.

Case #	p16	MUC6	Claudin18.2	PAX8	p53	ER	PR
1	-	+	-	-	+	-	-
2	-	+	++~+++	-	+	-	-
3	+	+	+++	-	-	-	-
4	+	+	+	-	+	-	-
5	-	+	+++	-	-	-	-
6	+	+	+++	-	-	-	-
7	+	+	+	-	-	-	-
8	+	+	++~+++	+	+	-	-
9	-	+	-	-	+	-	-
10	+	+	+	+	+	+~++	-
11	-	+	++	+	+	-	-
12	-	+	-	-	+	-	-

+, mildly positive; ++, moderately positive; +++, strongly positive, #: number.

## Data Availability

The datasets generated and/or analyzed during the current study are available from the corresponding authors upon reasonable request.

## Abbreviations

The following abbreviations are used in this manuscript:

GAS	gastric-type endocervical adenocarcinoma
SCC	squamous cell carcinoma
UEA	usual-type endocervical adenocarcinoma
FIGO	Federation of Gynecology and Obstetrics
AJCC	American Joint Committee on Cancer
PET/CT	positron emission tomography/computed tomography
SUV_max_	the max standardized uptake value
T/L SUV_max_	the ratio of cervical SUV_max_ to liver SUV_max_.
